# LDAPred: A Method Based on Information Flow Propagation and a Convolutional Neural Network for the Prediction of Disease-Associated lncRNAs

**DOI:** 10.3390/ijms20184458

**Published:** 2019-09-10

**Authors:** Ping Xuan, Lan Jia, Tiangang Zhang, Nan Sheng, Xiaokun Li, Jinbao Li

**Affiliations:** 1 School of Computer Science and Technology, Heilongjiang University, Harbin 150080, China; 2 Postdoctoral Program of Heilongjiang Hengxun Technology Co., Ltd., Harbin 150090, China; 3 School of Mathematical Science, Heilongjiang University, Harbin 150080, China

**Keywords:** lncRNA–disease association, information flow propagation, network topological structure, convolutional neural network, deep learning

## Abstract

Long non-coding RNAs (lncRNAs) play a crucial role in the pathogenesis and development of complex diseases. Predicting potential lncRNA–disease associations can improve our understanding of the molecular mechanisms of human diseases and help identify biomarkers for disease diagnosis, treatment, and prevention. Previous research methods have mostly integrated the similarity and association information of lncRNAs and diseases, without considering the topological structure information among these nodes, which is important for predicting lncRNA–disease associations. We propose a method based on information flow propagation and convolutional neural networks, called LDAPred, to predict disease-related lncRNAs. LDAPred not only integrates the similarities, associations, and interactions among lncRNAs, diseases, and miRNAs, but also exploits the topological structures formed by them. In this study, we construct a dual convolutional neural network-based framework that comprises the left and right sides. The embedding layer on the left side is established by utilizing lncRNA, miRNA, and disease-related biological premises. On the right side of the frame, multiple types of similarity, association, and interaction relationships among lncRNAs, diseases, and miRNAs are calculated based on information flow propagation on the bi-layer networks, such as the lncRNA–disease network. They contain the network topological structure and they are learned by the right side of the framework. The experimental results based on five-fold cross-validation indicate that LDAPred performs better than several state-of-the-art methods. Case studies on breast cancer, colon cancer, and osteosarcoma further demonstrate LDAPred’s ability to discover potential lncRNA–disease associations.

## 1. Introduction

Many studies have indicated that protein-coding genes only account for ~2% of the human genome, whereas non-coding protein sequences account for ~98% [1,2,3,4,5]. Non-coding RNA, especially long non-coding RNA with a length exceeding 200 nucleotides (lncRNA), plays an important role in various biological processes, such as transcription, translation, epigenetic regulation, splicing, differentiation, the immune response, and cell cycle control. Mutations and disorders of lncRNA are associated with a variety of human diseases [6,7,8,9]. For example, lncRNA PCA3 is a biomarker for potential cancer diagnosis because it is associated with normal tissues and increases the expression level of prostate tumors by 60× [10,11]. Therefore, it is necessary to discover more potential lncRNA–disease associations to understand the molecular mechanism of human disease at the lncRNA level and to promote the diagnosis of diseases and identification of related biomarkers.

The calculation methods employed for predicting potential lncRNA–disease associations can be broadly divided into three categories. The first method uses the biological information of lncRNA to identify lncRNA–disease associations, such as the expression profile, tissue specificity, and genome location. Li et al. [12] predicted lncRNA–disease associations based on known gene–disease associations through the neighborhood relationship between lncRNA and genome-located genes. However, their model only applies to a small number of lncRNAs. Liu et al. [13] identified a potential association by combining the tissue specificity of lncRNA with the co-expression of gene–lncRNA associations. Chen et al. [14] integrated the lncRNA expression profile, functional similarity of lncRNA, known lncRNA–disease associations, the semantic similarity of disease, and the Gaussian cross-configuration kernel information to determine the potential association between lncRNA and diseases. However, this method suffers from low tissue-specific expression and limited lncRNA expression information.

The second method uses machine learning models to predict the potential associations. Chen et al. [15] proposed a Laplace regularization least square method (LRLSLDA), which uses semi-supervised learning to identify lncRNA–disease associations. However, this method uses classifiers, and it often fails to achieve acceptable results because of the unbalanced distribution of these classifiers. Lan et al. [16] used the bagging support vector machine (SVM) classifier and fused different data sources to predict potential associations between lncRNAs and diseases. However, this method cannot effectively fuse lncRNAs from different cores 

The third method establishes heterogeneous networks, based on which lncRNA–disease associations can be predicted. Zhang et al. [17] constructed a heterogeneous network containing lncRNA, protein, and disease information and obtained a disease-related candidate lncRNA by disseminating the information flow in the heterogeneous network. Yao et al. [18] constructed a multi-level heterogeneous network by integrating lncRNA, genes, and phenotypes, and designed a heterogeneous random walk on this network. There are also several methods for random walk on lncRNA networks, with similar functions or heterogeneous networks composed of lncRNA, genes, and diseases to infer candidate lncRNAs [18,19,20,21]. Xuan et al. [22] established lncRNA, miRNA, and isomerism networks to predict the potential association between lncRNA and diseases, considering the attention mechanism. The deep learning methods have also been applied to predict disease-related lncRNAs [23,24]. However, most of these research methods learn the information between nodes without considering the network topology between the nodes. Therefore, prediction methods integrating network topology information are expected to exhibit a better performance.

In this study, we propose a method, called LDAPred, based on information flow propagation and a convolutional neural network, to predict potential lncRNA–disease associations. LDAPred utilises the similarities, associations, and interactions among lncRNAs, miRNAs, and diseases. On the left side of the network, the original feature matrix of lncRNA–disease node pairs was constructed from the biological perspective. On the right side, according to the information flow propagation of the bi-layer network formed by lncRNA, miRNA, and disease, the possibility of interconnections between them was calculated, and the characteristic matrix was formed. Dual convolution was used to learn deeper features and make association predictions. Combined with five-fold cross-validation experiments, the results indicate that LDAPred is better than several existing methods for the prediction of candidate lncRNAs. Moreover, the results of the case study on breast cancer, colon cancer, and osteosarcoma also indicate that LDAPred has a strong ability to identify potential disease lncRNAs.

## 2. Result and Discussion

### 2.1. Parameter Settings

To achieve the best prediction result, we repeatedly verified the results by conducting experiments. Finally, the filter used in the convolutional layer and the pooling layer in the dual channel system was set to the dimension of 2×2. The convolution process of the two channels was consistent. We set the number of the first layer filters $n_{conv1}$ and $n_{conv3}$ of the left and right convolution modules as 8, and the number of the second layer filters $n_{conv2}$ and $n_{conv4}$ of the left and right channels as 16. In the right embedding, hyperparameter γ, which was used to balance the proportion of one-hop and two-hop information, was set to 0.2. Finally, we balanced the score ratio of the two paths by using the parameter λ=0.7.

### 2.2. Evaluation Metrics

To evaluate the performance of the prediction model, we used five-fold cross-validation. First, the known 2687 lncRNA–disease associations were divided into five groups, four of which were used as the training set and one as the test set. Second, we deleted the association in the test set when calculating the similarity of lncRNAs. We regarded those with lncRNA-related diseases in the test set as positive cases and those without any association as negative cases.

After using our prediction model to evaluate the associated scores of the test samples, the scores of the samples were ranked in descending order. The higher the ranking of the positive examples, the better the prediction performance of the model. We measured the global performance of our prediction model by drawing the receiver operating characteristic (ROC) curve and calculating the area under the curve (AUC). The true positive rate (TPR) and false positive rate (FPR) can be defined as follows:

(1) $$\mathrm{TPR}=\frac{\mathrm{TP}}{\mathrm{TP}+\mathrm{FN}},\;\mathrm{FPR}=\frac{\mathrm{FP}}{\mathrm{TN}+\mathrm{FP}}$$

where *TP* is the number of positive samples that are considered positive, and *TN* is the number of counterexamples that are considered counterexamples. *FN* is the number of positive examples that are considered counterexamples, and *FP* is the number of counterexamples that are considered positive examples. Finally, the average of all disease AUCs was taken to represent the performance of the predictive model. The higher the value, the higher the global performance of the model.

Because the lncRNA–disease sample has a number of associated positive examples that are smaller than the unrelated or unrecognized counterexamples, there is a serious imbalance ratio. Therefore, we also used the precision–recall (PR) curve to measure the overall performance of the model. The larger the area under the PR curve (AUPR), the better the prediction performance. The precision and recall can be calculated as follows:

(2) $$\mathrm{precision}=\frac{\mathrm{TP}}{\mathrm{TP}+\mathrm{FP}},\;\mathrm{recall}=\frac{\mathrm{TP}}{\mathrm{TP}+\mathrm{FN}}.$$

Biological experiments are costly and time-consuming and limited by equipment precision and human error; thus, biologists choose to predict the top lncRNA to verify the disease associated with it. Therefore, we also calculated the recall rate of the first *k* (30, 60, 90, ..., 240) samples, i.e., the ratio of the positive samples in the first *k* samples to all the predicted positive samples, as another performance index.

### 2.3. Comparison with Other Methods

To reveal the advantages of considering network topology information in lncRNA–disease association prediction modeling and demonstrate the strong performance of our model, we selected four latest lncRNA–disease association prediction methods, namely SIMCLDA [25], Ping’s method [26], MFLDA [27], and LDAP [16], for comparison. To make a fair comparison, we used the optimal values recommended in these articles as superparameters of the four methods.

**Figure 1:**
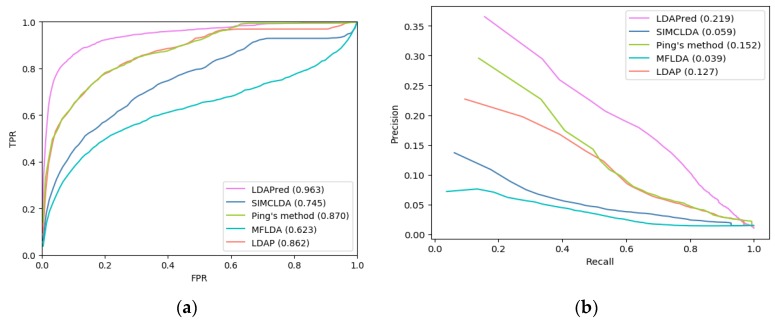
(**a**) Receiver operating characteristic (ROC) curves of LDAPred and the other four methods. (**b**) Precision–recall (PR) curves of LDAPred and the other four methods.

As shown in Figure 1a, our method, LDAPred, achieved the best performance in all 405 diseases; i.e., the average area under the ROC curve was 0.963. This is 21.8% higher than that of SIMCLDA, 9.3% higher than that of Ping’s method, 34% higher than that of MFLDA, and 10.1% higher than that of LDAP. We also listed five methods for AUCs for 10 well-characterized diseases (Table 1). Each disease was associated with at least 15 lncRNAs. Table 1 shows that LDAPred performs best for 8 out of 10 diseases. Both Ping’s method and LDAP achieved a good performance with similar ROC values as they both used the similarity calculated from different angles of lncRNA and disease. The performance of MFLDA is the worst of the five methods because it does not consider the similarity of the disease and lncRNA during the prediction process. LDAPred has the best performance among the five methods because it considers the network topology among lncRNA, disease, and miRNA, and learns the depth representation of these topologies.

As shown in Figure 1b and Table 2, the average PR curve of LDAPred for 405 diseases was higher than that of the other four methods. The average AUPR (area under PR curves) of our method’s PR curve is 0.219, which is higher than those of SIMCLDA, Ping’s method, MFLDA, and LDAP (19%, 6.7%, 18%, and 9.2%, respectively). Of the 10 diseases with clear characteristics associated with lncRNA, LDAPred performed the best for 6 diseases. 

In addition, to assess whether the AUC performance of LDAPred for all 405 diseases is better than those of the other four methods, we performed a paired Wilcoxon test. The statistical results are shown in Table 3. For AUC and AUPR, LDAPred performed significantly better than all the other methods at a *p*-value of 0.05. 

**Figure 2:**
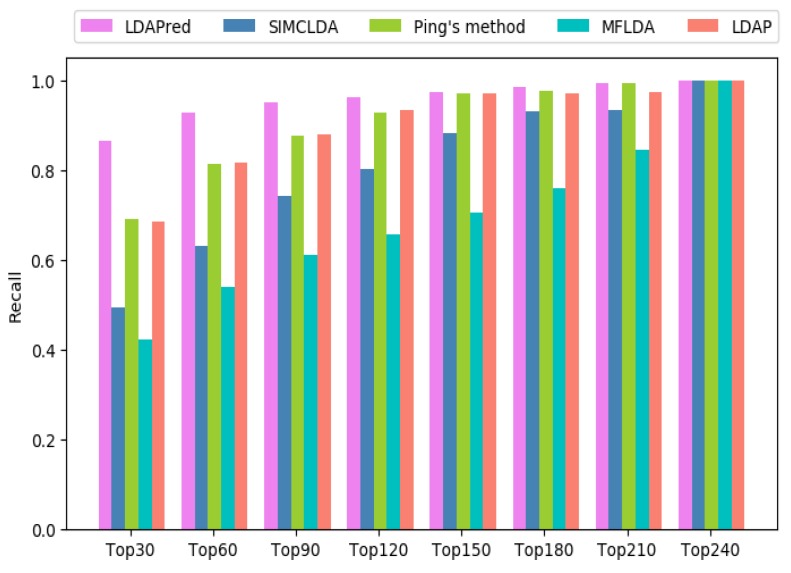
Recall values of the top *k* candidates of LDAPred and the other four methods.

The higher the recall rate of the top *k* lncRNAs, the greater the number of correctly identified lncRNAs that are related to the disease. Figure 2 shows the average recall rate for the first *k* samples of all 405 diseases. LDAPred is superior to the other methods at different *k* values, accounting for 86.4% in the top 30, 92.8% in the top 60, 95.1% in the top 90, and 96.3% in the top 120. The recall rate of Ping’s method is very close to that of LDAP. The former accounts for 68.9%, 81.2%, 87.5%, and 92.7% among the top 30, 60, 90, and 120, whereas the latter accounts for 68.5%, 81.7%, 88.0%, and 93.3%, respectively. SIMCLDA accounts for 49.3% in the top 30, 63.0% in the top 60, 74.1% in the top 90, and 80.3% in the top 120, exhibiting lower values than Ping’s method and LDAP. Compared to the four methods, MFLDA always shows the worst performance, accounting for 42.0%, 53.9%, 60.9%, and 65.5%, respectively.

### 2.4. Case Studies on Breast Cancer, Colon Cancer, and Osteosarcoma

To further demonstrate the LDAPred’s ability to detect disease-related lncRNAs, we used two separate databases (Lnc2Cancer and lncRNADisease) and related literature to validate candidate genes for breast cancer, colon cancer, and osteosarcoma. The top 15 candidate lncRNAs associated with these cancers were analysed separately (Table 4).

Lnc2Cancer is an experimentally supported lncRNA manual management database for various human cancers [28]. It contains more than 1500 published papers collected by hand and 1057 interactions extracted from 531 lncRNAs and 86 cancers, i.e., the expression level (up or down) of lncRNA in cancer [29]. The LncRNADisease 2.0 database is not only a resource that curates the experimentally-supported lncRNA–disease association data, but also a platform that integrates tools for predicting novel lncRNA–disease associations. We used lncRNADisease and lncRNADisease_P to demonstrate the association between experimental support and prediction, respectively. As shown in Table 4, Lnc2Cancer contains 14 candidate lncRNAs, and lncRNADisease contains 13 candidate lncRNAs, confirming the association. lncRNADisease_P contains 23 candidate lncRNAs, confirming that these lncRNAs are more likely to be associated with the diseases.

The remaining three candidates reported in previous studies are marked as the “literature” in Table 4. Among them, the expression of LATS2 is often down-regulated in breast cancer, and the oncogenic function of LINC00673 is determined in part by inhibiting the expression of KLF2 and LATS2 [30]. MEG8 can directly interact with the epigenetic mechanism and may have a predictive effect on the prognosis of breast cancer [31]. In the PWAR5 prediction experiment, the factors that affect the mother cell tumor also affect the breast cancer. These three candidates may be involved in the progression of breast cancer. Another candidate is represented by GEO in Table 4. The GEO Dataset is a relatively comprehensive public gene expression database, and it indicates that NPSR1-AS1 is associated with colon cancer recurrence [32]. The remaining four are labeled as “Unconfirmed” candidates, indicating that they are not in the database or in the related literature. Case studies of these three diseases confirm that LDAPred has a strong ability to detect lncRNAs with potential diseases.

## 3. Materials and Methods

### 3.1. Dataset

To predict the relationships between lncRNAs and diseases, we needed to integrate the attributes and characteristics of each node of the lncRNAs, miRNAs, and diseases. Therefore, we downloaded 2687 lncRNA–disease associations from the LncRNADisease [33] and Lnc2Cancer [28] databases and from the lncRNAs functional description database, GeneRIF [34]. We calculated the similarity of 249 lncRNAs based on the diseases associated with lncRNAs. We obtained the interaction data of 1002 lncRNAs and miRNAs from starBase v2.0, an open source platform containing multiple RNA interactions [35]. We downloaded 13,559 miRNA and disease associations from HMDD v1.0 [36], a human miRNA and disease association database supported by experiments. We calculated the similarity of 495 miRNAs based on the disease association of miRNA. Finally, we downloaded the similarity data of 405 diseases from DincRNA v1.0 [37], calculated based on the directed myelogram of the diseases.

### 3.2. Similarity Calculation and Data Representation

#### 3.2.1. Semantic Similarity of Diseases

A disease can be expressed as a directed acyclic graph (DAG), which can be obtained from Medical Subject Headings (MeSH), and it includes all relevant annotated items of the disease. Studies have shown that the more common the DAG of two diseases, the more similar the two diseases. Wang et al. [38] measured the semantic similarity between diseases according to the DAG of the disease. In this study, we used the calculated semantic similarity of the disease. We utilised matrix $D\in {ℜ}^{n_{d}\times n_{d}}$ to represent the similarity of the diseases, where $n_{d}$ is the number of diseases, $D_{ij}$ denotes the similarity between diseases $d_{i}$ and $d_{j}$, and the similarity value changes between 0 and 1.

#### 3.2.2. Similarity of lncRNAs

The more similar the functions of two lncRNAs, the more similar the related diseases. Therefore, we calculated the similarity of two lncRNAs by calculating the similarity of the two lncRNA-associated diseases. For example, lncRNA $l_{a}$ is associated with diseases $d_{1}$, $d_{3}$, $d_{4}$, and $d_{6}$, and lncRNA $l_{b}$ is associated with diseases $d_{1}$, $d_{3}$, and $d_{4}$. Using the method of Xuan et al. [22], the similarity between $S_{a}=\left\{d_{1},d_{3},d_{4},d_{6}\right\}$ and $S_{b}=\left\{d_{1},d_{2},d_{3}\right\}$ was calculated, and the calculation result was taken as the similarity between $l_{a}$ and $l_{b}$. We used a similarity matrix $L\in {ℜ}^{n_{l}\times n_{l}}$ to represent the similarity of lncRNAs, where $n_{l}$ is the number of lncRNAs, and $L_{ij}$ represents the similarity between lncRNA $l_{i}$ and lncRNA $l_{j}$, with similarity values varying between 0 and 1.

#### 3.2.3. Similarity of miRNAs

Similar to the lncRNA similarity calculation, the miRNA similarity was calculated based on the associated diseases. We used the matrix $M\in {ℜ}^{n_{m}\times n_{m}}$ to represent the similarity of miRNAs, where $n_{m}$ is the number of miRNAs, $M_{ij}$ represents the similarity between miRNA $m_{i}$ and miRNA $m_{j}$, and the similarity values are distributed between 0 and 1.

#### 3.2.4. Interaction Matrix

In this study, heterogeneous data resources were synthesized and the interaction matrix was established: the lncRNA–disease association matrix $A\in {ℜ}^{n_{l}\times n_{d}}$, lncRNA–miRNA interaction matrix $B\in {ℜ}^{n_{l}\times n_{m}}$, and miRNA–disease association matrix $C\in {ℜ}^{n_{m}\times n_{d}}$. In matrix *A*, $n_{l}$ is the number of lncRNAs and $n_{d}$ denotes the number of diseases. If lncRNA $A_{i}$ is associated with disease $A_{j}$, then $A_{ij}$ is 1; if there is no association, then $A_{ij}$ is 0. In matrix *B*, $n_{l}$ is the number of lncRNAs and $n_{m}$ represents the number of miRNAs. If lncRNA $B_{i}$ is associated with disease $B_{j}$, then $B_{ij}$ is 1; if there is no association, then $B_{ij}$ is 0. In matrix *C*, $n_{m}$ is the number of miRNAs and $n_{d}$ is the number of diseases. If miRNA $C_{i}$ is associated with disease $C_{j}$, then $C_{ij}$ is 1; otherwise, $C_{ij}$ is 0.

### 3.3. LncRNA–Disease Association Prediction Model Based on a Dual Convolutional Neural Network

We constructed a dual convolutional neural network (CNN) predictive model to predict the lncRNA–disease associations. The left side uses the original information of the lncRNA $l_{i}$ and disease $d_{j}$ node pair to learn its original representation. The right side learns the path association representation of $l_{i}$ and $d_{j}$ from the network topology structure and information flow propagation. Then, the two representations are combined by a CNN and the complete connection layer to obtain the final association prediction score of $l_{i}$ and $d_{j}$ for the association prediction of $l_{i}$ and $d_{j}$, respectively.

#### 3.3.1. Embedded Layer

##### Establishment of the Left Feature Matrix

**Figure 3:**
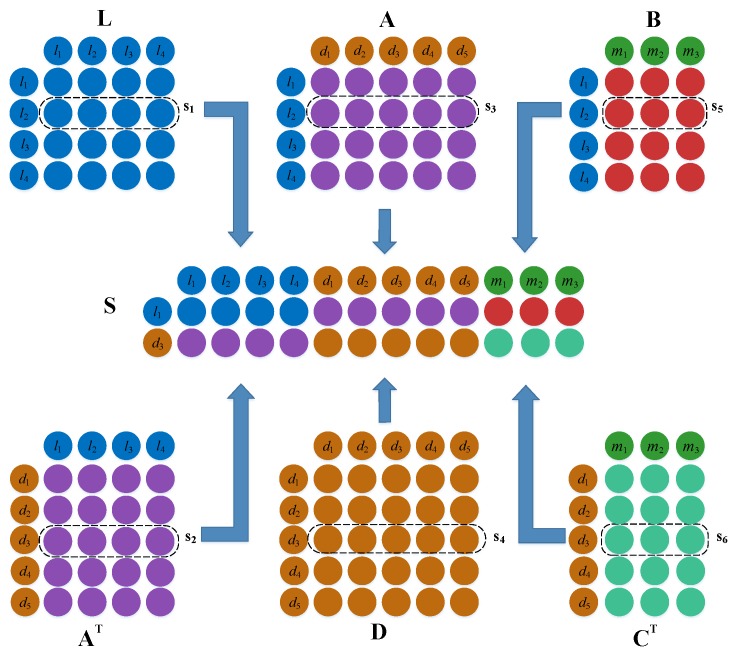
Construction of the original eigenmatrix of lncRNA *l*_2_ and disease *d*_3_.

We utilized lncRNA $l_{2}$ and disease $d_{3}$ as examples to describe the establishment of the feature matrix. First, if $l_{2}$ and $d_{3}$ have a connection with more identical lncRNAs, then $l_{2}$ and $d_{3}$ are more likely to be associated. Therefore, we took the similarity vector $s_{1}\in L$ between lncRNA $l_{2}$ and all lncRNAs, which comprise the second row of matrix *L*, and the association vector $s_{2}\in A$ between disease $d_{3}$ and all lncRNAs, which comprise the third column of matrix *A*, and combined them together. Second, if $l_{2}$ and $d_{3}$ have a relationship with more of the same disease, then $l_{2}$ and $d_{3}$ are more likely to be associated. Therefore, we combined the second row of matrix A with the second row of matrix D, which is the $l_{2}$-associated vector $s_{3}\in A$ for all diseases, and the similarity vector $s_{4}\in D$ for disease $d_{3}$ with all diseases. Third, if $l_{2}$ and $d_{3}$ are associated with more of the same miRNA, then $l_{2}$ and $d_{3}$ are more likely to be associated. Therefore, we took vector $s_{5}\in B$, for which $l_{2}$ interacts with all miRNAs; i.e., the second row of matrix B and the third column of matrix *C*, vector $s_{6}\in$ *C*, and $d_{3}$ associated with all miRNAs. Finally, we stitched these vector combinations into the feature matrix $S=\left\{s_{1},s_{2},s_{3},s_{4},s_{5},s_{6}\right\}\in {ℜ}^{2\times \left(n_{d}+n_{l}+n_{m}\right)}$ (Figure 3).

##### Establishment of the Right Side Topological Information Matrix

Inspired by Chen et al. [14], we constructed a comprehensive matrix $T=\left\{t_{1},t_{2},t_{3},t_{4},t_{5},t_{6}\right\}\in {ℜ}^{2\times \left(n_{d}+n_{l}+n_{m}\right)},$ which further considers the topological structure of lncRNA, miRNA, and disease-related bi-layer networks via information flow propagation.

In a network comprising lncRNAs, *L* represents the original information between lncRNA nodes; i.e., the one-hop similarity information. L×L represents the similarity of lncRNA nodes after two hops, and γ is a hyperparameter, which balances the proportion of one hop and two hops and ranges from 0 to 1. $L^{\prime }$ is used to integrate the one hop and two hop similarity information in the path. $L_{ij}^{\prime }$ represents the similarity value of lncRNAs $l_{i}$ and $l_{j}$ after integrating the topological information. $L^{\prime }$ is calculated as follows:

(3) $$L^{\prime }=\gamma \cdot L+\gamma^{2}\left(L\cdot L\right).$$

Similarly, $D^{\prime }$ integrates the one hop and two hop similarity information of the disease, and $D_{ij}^{\prime }$ is the similarity between diseases $d_{i}$ and $d_{j}$ after integrating the information flow. The calculation of $D^{\prime }$ is as follows:

(4) $$D^{\prime }=\gamma \cdot D+\gamma^{2}\left(D\cdot D\right).$$

In a network comprising lncRNAs and diseases, A represents the one-hop information between lncRNA and disease node pairs, and (L·A+A·D) represents the degree of association between lncRNA and disease node pairs after two hops. γ is a hyperparameter that balances the proportion of one hop and two hops and ranges from 0 to 1. $A^{\prime }$ represents the similarity after integrating the path information, and $A_{ij}^{\prime }$ is the ratio of lncRNA $l_{i}$ and disease $d_{j}$ after two hops. The degree of association $A^{\prime }$ is calculated as shown in Equation (5),

(5) $$A^{\prime }=\gamma \cdot A+\gamma^{2}\left(L\cdot A+A\cdot D\right).$$

Similarly, the association information between the disease and miRNA is expressed by ${\left(C^{T}\right)}^{\prime }$, and the calculation process is expressed by Equation (6).

(6) $${\left(C^{T}\right)}^{\prime }=\gamma \cdot C^{T}+\gamma^{2}\left(D\cdot C^{T}+C^{T}\cdot M\right).$$

${\left(A^{T}\right)}^{\prime }$ is a transposition of $A^{\prime }$, indicating the association between the disease and lncRNA by information flow propagation bi-layer networks, and Equation (7),

(7) $${\left(A^{T}\right)}^{\prime }=\;\gamma \cdot A^{T}+\gamma^{2}\left(D\cdot A^{T}+A^{T}\cdot L\right),$$

indicates the calculation process.

In the network composed of lncRNA and miRNA, B represents the original interaction information between lncRNA and miRNA node pairs, i.e., the one-hop information, and (L·B+B·M) represents the degree of association information after two hops. γ is used to balance the proportion of one hop and two hops. The one-hop and two-hop integration information is represented by $B^{\prime }$, and $B_{ij}^{\prime }$ is used to represent the degree of association between lncRNA $l_{i}$ and miRNA $m_{j}$ with the bi-layer network information. $B^{\prime }$ is calculated as follows:

(8) $$B^{\prime }=\gamma \cdot B+\gamma^{2}\left(L\cdot B+B\cdot M\right).$$

**Figure 4:**
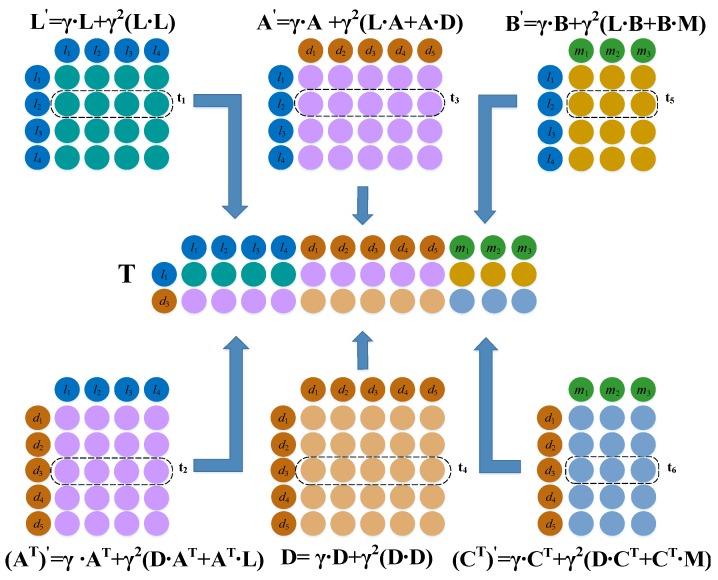
Construction of the topological feature matrix of lncRNA *l*_2_ and disease *d*_3_.

Finally, we took the second row of matrix $L^{\prime }$ as vector $t_{1}$, the third row of matrix ${\left(A^{T}\right)}^{\prime }$ as vector $t_{2}$, the second line of matrix $A^{\prime }$ as vector $t_{3}$, the third line of matrix $D^{\prime }$ as vector $t_{4}$, the second line of matrix $B^{\prime }$ as vector $t_{5}$, and the third row of matrix ${\left(C^{T}\right)}^{\prime }$ as vector $t_{6}$. We spliced the combination of these vectors into the path eigenmatrix $T=\left\{t_{1},t_{2},t_{3},t_{4},t_{5},t_{6}\right\}\in {ℜ}^{2\times \left(n_{d}+n_{l}+n_{m}\right)}$ as the right embedding matrix (Figure 4).

#### 3.3.2. Convolution Module

Because the left and right convolution processes are similar, we will only describe the left convolution process in detail herein. $S=\left\{s_{1},s_{2},s_{3},s_{4},s_{5},s_{6}\right\}\in {ℜ}^{2\times \left(n_{d}+n_{l}+n_{m}\right)}$ was used as the left input of the CNN module. In the first convolution, the length and width of the convolution filter were respectively set to $w_{f}$ and $w_{d}$, and the number of convolution filters was set to $n_{conv1}$, which can be expressed as $W_{conv1}\in {ℜ}^{w_{f}\times w_{d}\times n_{conv1}}$. We applied filter $W_{conv1}$ to *S*. In addition, to fully learn the edge information, we applied wide convolution by padding zeros before convolution. The definitions of $S_{k,i,j}$ and $M_{conv1,k}$ are as follows:

(9) $$S_{k,i,j}=S\left(i:i+w_{d},j:j+w_{f}\right),\;S_{k,i,j}\in {ℜ}^{w_{f}\times w_{d}}$$

(10) $$M_{conv1,k}\left(i,j\right)=f(W_{conv1}\left(k,:,:\right)\times S_{k,i,j}+b_{conv1}\left(k\right)),\;i\in ⎣1,S_{l}+2-w_{f}+1⎦,\;\;j\in \left[1,S_{w}+2-w_{d}+1\right],\;k\in \left[1,n_{conv1}\right],$$

where S(i,j) is the *i*th row and *j*th column element of the embedded layer *S*, and $S_{k,i,j}$ is the region within the filter when the *k*th filter is slid to position S(i,j). f is the rectified linear unit (ReLU) activation function, and $b_{conv1}\in {ℜ}^{n_{conv1}}$ is the offset term. The output feature, which is the result after convolution, is $M\in {ℜ}^{n_{conv1}\times \left(S_{w}+3-n_{d}\right)\times \left(S_{l}+3-n_{f}\right)}$.

In the pooling layer, $M_{conv1}$ performs a max pooling operation; i.e., the output in each sub-area is the maximum value. The pooling layer can reduce the length of the feature graph output of convolution and the number of parameters of the model. The pooling operation can be expressed as follows:

(11) $$M_{conv1}\left(k\right)=\max\;\left(M\left(k\right)\right),\;k\in \left[1,n_{conv1}\right].$$

After two convolutions and pooling were completed, we obtained the final representation $M_{conv2}\left(k\right)$, $k\in \left[1,n_{conv2}\right]$, which represents the number of filters, where $n_{conv2}$ is the number of filters for the second convolution.

Finally, we flattened $M_{conv2}$ and obtained the association prediction scores of $l_{2}$ and $d_{3}$ through the fully connected layer. The score $score_{1}$ can be defined as

(12) $$score_{1}=H\times M_{conv2},$$

where *H* is the weight matrix between the fully connected layer and the output layer, and $score_{1}\in {ℜ}^{2\times 1}$ represents the matrix evaluated as the associated score and the unassociated score. We used the $score_{1}$ as the predicted association score of $l_{2}$ and $d_{3}$.

Similarly, we employed $T=\left\{t_{1},t_{2},t_{3},t_{4},t_{5},t_{6}\right\}\in {ℜ}^{2\times \left(n_{d}+n_{l}+n_{m}\right)}$ as the input to the right CNN module and obtained the output of the second pooling layer. $N_{conv4}$ is the number of filters. The associated prediction scores of $l_{2}$ and $d_{3}$ were obtained through the fully connected layer. The score can be defined as follows:

(13) $$score_{2}=K\times N_{conv4},$$

where *K* is the weight matrix between the fully connected layer and the output layer, and $score_{2}$ is the associated prediction score.

#### 3.3.3. Dual Combination Strategy

To fully utilize the dual prediction score matrix, we designed a dual combination strategy to train the model and obtain the final prediction score. We used λ∈[0,1] to balance the weight of the two paths, and the final predicted score was expressed by the score, which can be defined as follows:

(14) $$score=\lambda \times score_{1}+\left(1-\lambda \right)\times score_{2}.$$

**Figure 5:**
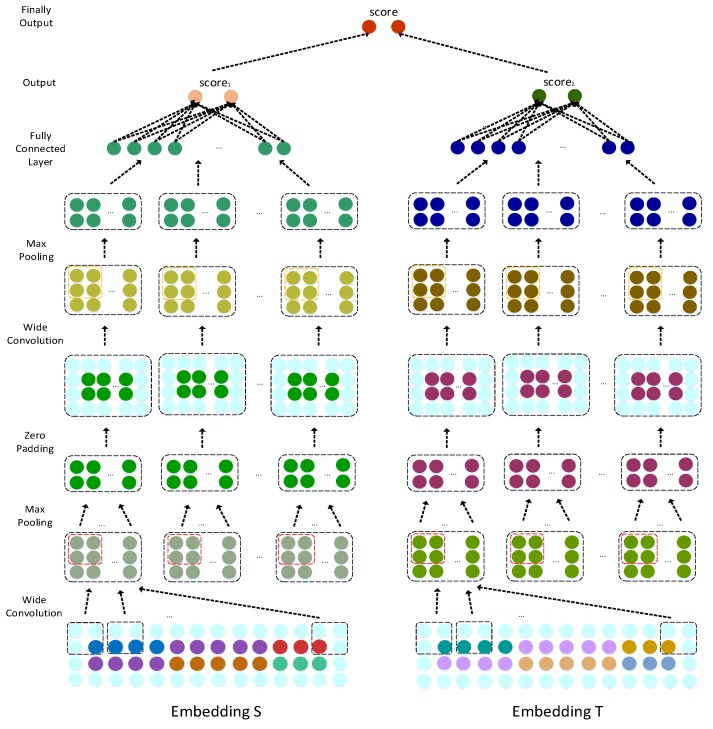
lncRNA–disease association prediction framework based on a dual CNN.

The loss functions of the left and right CNNs can be defined as

(15) $$l_{1}=-\sum_{i=1}^{M}\left[y_{label}\times loga+\left(1-y_{label}\right)\times log\left(1-a\right)\right],\;a=\frac{e^{score_{1}}}{\sum_{j=1}^{2}e^{score_{1}\left(j\right)}}$$

(16) $$l_{2}=-\sum_{i=1}^{M}\left[y_{label}\times logb+\left(1-y_{label}\right)\times log\left(1-b\right)\right],\;b=\frac{e^{score_{2}}}{\sum_{j=1}^{2}e^{score_{2}\left(j\right)}}$$

where $y_{label}$ represents the actual association label between lncRNA and the disease. When lncRNA is associated, it is 1; otherwise, $y_{label}$ is 0. $score_{1}$ and $score_{2}$ represent $l_{2}$ and $d_{3}$, which are the associated scores. *M* represents the number of training samples, and a and b represent the probabilities obtained by the Softmax function. The dual convolution and combining processes are displayed in Figure 5. The top 50 potential lncRNA candidates for 405 diseases are listed in supplementary Table S1.

## 4. Conclusions

LDAPred, which is a new method based on a dual convolutional neural network, was developed to predict the potential associations between lncRNAs and diseases. According to the biological premise that lncRNAs are likely to possess associations with diseases, the embedding layer was established from a biological perspective. The left and right embedding layers capture the original similarities, associations, and interactions among lncRNAs, miRNAs, and diseases, as well as the topological structures of bi-layer networks. The original representation of lncRNA–disease pairs and their network representations were learned by the new framework based on dual convolutional neural networks and information flow propagation. Cross-validation results for 405 diseases and case studies on three diseases indicated that LDAPred has a strong ability to predict potential associations between lncRNAs and diseases.

## Figures and Tables

**Table 1:** Area under ROC curves (AUC) of LDAPred and other methods for all diseases and 10 well-characterized diseases.

Disease Name	Percentage of Disease-Related lncRNAs	AUC				
		LDAPred	SIMCLDA	Ping’s Method	MFLDA	LDAP
Respiratory system cancer	1.1%	**0.913**	0.789	0.911	0.719	0.891
Organ system cancer	1.6%	**0.958**	0.820	0.950	0.729	0.884
Intestinal cancer	2.3%	**0.963**	0.811	0.909	0.559	0.905
Prostate cancer	1.0%	**0.951**	0.873	0.826	0.553	0.711
Lung cancer	1.1%	0.833	0.790	**0.911**	0.676	0.883
Breast cancer	0.1%	**0.970**	0.742	0.871	0.517	0.830
Reproductive organ cancer	1.1%	**0.993**	0.707	0.818	0.741	0.742
Gastrointestinal system cancer	0.1%	**0.985**	0.784	0.896	0.582	0.867
Liver cancer	1.5%	**0.911**	0.799	0.910	0.634	0.898
Hepatocellular carcinoma	1.5%	0.867	0.765	**0.903**	0.688	0.902

The bold values indicate the higher AUCs.

**Table 2:** Area under PR curves (AUPR) of LDAPred and other methods for all diseases and 10 well-characterized diseases.

Disease Name	AUPR				
	LDAPred	SIMCLDA	Ping’s Method	MFLDA	LDAP
Respiratory system cancer	0.178	0.149	**0.414**	0.072	0.303
Organ system cancer	0.029	0.411	**0.765**	0.338	0.628
Intestinal cancer	**0.271**	0.141	0.252	0.042	0.246
Prostate cancer	**0.338**	0.176	0.333	0.095	0.297
Lung cancer	**0.655**	0.138	0.334	0.008	0.094
Breast cancer	0.125	0.445	**0.803**	0.476	0.629
Reproductive organ cancer	**0.490**	0.047	0.403	0.031	0.396
Gastrointestinal system cancer	**0.500**	0.130	0.271	0.104	0.238
Liver cancer	**0.672**	0.201	0.526	0.086	0.498
Hepatocellular carcinoma	0.198	0.096	**0.239**	0.082	0.303

The bold values indicate the higher AUPRs.

**Table 3:** Results of a paired Wilcoxon-test for LDAPred and four other contrast methods in terms of AUCs and AUPRs.

*p*-Value and Other Methods	SIMCLDA	Ping’s Method	MFLDA	LDAP
*p*-values of AUCs	2.4816 × 10^−^^17^	0.0079 × 10^−15^	1.2144 × 10^−15^	0.0033 × 10^−14^
*p*-values of AUPRs	0.0118 × 10^−14^	0.3000 × 10^−13^	0.0030 × 10^−14^	0.9211 × 10^−11^

**Table 4:** Candidate long non-coding RNAs (lncRNAs) associated with breast cancer, colon cancer, and osteosarcoma.

Disease Name	Rank	LncRNA Name	Description	Rank	LncRNA Name	Description
Breast cancer	1	AFAP1-AS1	Lnc2Cancer, lncRNADisease	9	CECR7	Unconfirmed
	2	LINC00675	Literature	10	DBET	lncRNADisease_P
	3	H19	Lnc2Cancer, lncRNADisease_P	11	CARMN	lncRNADisease_P
	4	HOTTIP	Lnc2Cancer, lncRNADisease_P	12	DISC1FP1	lncRNADisease_P
	5	HCG9	lncRNADisease_P	13	VLDLR-AS1	lncRNADisease_P
	6	MEG8	Literature	14	PWAR5	Literature
	7	LINC00315	lncRNADisease_P	15	LINC00479	lncRNADisease_P
	8	GABPB1-AS1	Unconfirmed			
Colon cancer	1	NPSR1-AS1	GEO	9	LINC00477	lncRNADisease_P
	2	MEG3	Lnc2Cancer, lncRNADisease	10	PARD6G-AS1	lncRNADisease_P
	3	H19	Lnc2Cancer, lncRNADisease	11	OIP5-AS1	lncRNADisease_P
	4	CCAT2	Lnc2Cancer, lncRNADisease	12	LINC01184	lncRNADisease_P
	5	HOTAIR	Lnc2Cancer, lncRNADisease	13	CARMN	lncRNADisease_P
	6	CCAT1	Lnc2Cancer, lncRNADisease	14	MEG8	lncRNADisease_P
	7	MALAT1	Lnc2Cancer, lncRNADisease	15	GABPB1-AS	lncRNADisease_P
	8	GATA3-AS1	lncRNADisease_P			
Osteosarcoma	1	HOTAIR	Lnc2Cancer, lncRNADisease	9	MEG8	lncRNADisease_P
	2	LINC00673	Lnc2Cancer, lncRNADisease	10	GNAS-AS1	lncRNADisease_P
	3	MIR17HG	lncRNADisease_P	11	PTCSC2	lncRNADisease_P
	4	HULC	Lnc2Cancer, lncRNADisease_P	12	LINC00319	Unconfirmed
	5	TUSC7	Lnc2Cancer, lncRNADisease	13	GABPB1-AS1	Unconfirmed
	6	HOTTIP	Lnc2Cancer, lncRNADisease	14	LINC00473	Lnc2Cancer, lncRNADisease_P
	7	MEG3	Lnc2Cancer, lncRNADisease	15	VLDLR-AS1	lncRNADisease
	8	BANCR	Lnc2Cancer, lncRNADisease			

## Supplementary Materials

Supplementary Materials can be found at https://www.mdpi.com/1422-0067/20/18/4458/s1. Table S1: The top 50 potential lncRNA candidates for 405 diseases.
