# Supplementary material for: LDAPred: A Method Based on Information Flow Propagation and a Convolutional Neural Network for the Prediction of Disease-Associated lncRNAs
Source: Int J Mol Sci. 2019 Sep 10;20(18):4458. doi: 10.3390/ijms20184458 (PMC6771133; doi:10.3390/ijms20184458)
Supplement: Supplementary file 1 [file ijms-20-04458-s001.zip › LDAPred_Certificate_of_editing.pdf]

CERTIFICATE OF ENGLISH EDITING

This document certifies that the paper listed below has been edited to ensure that the language is clear and free of errors. The logical presentation of ideas and the structure of the paper were also checked during the editing process. The edit was performed by professional editors at Editage, a division of Cactus Communications. The intent of the author's message was not altered in any way during the editing process. The quality of the edit has been guaranteed, with the assumption that our suggested changes have been accepted and have not been further altered without the knowledge of our editors.

Title Of The Paper

LDAPred

Authors

Ping Xuan 1, Lan Jia 1, Tiangang Zhang 2,*, Nan Sheng 1, Xiaokun Li3 and Jinbao Li 1

Job Code

BCYEE_1

editage by CACTUS

Signature

Vikas Narang

Vikas Narang,
Senior Vice President,
Operations-Author Services, Editage

Date of Issue
June 22, 2019

Editage, a brand of Cactus Communications, offers professional English language editing and publication support services to authors engaged in over 500 areas of research. Through its community of experienced editors, which includes doctors, engineers, published scientists, and researchers with peer review experience, Editage has successfully helped authors get published in internationally reputed journals. Authors who work with Editage are guaranteed excellent language quality and timely delivery.

INFORMATION SECURITY MANAGEMENT SYSTEM DNV·GL ISO/IEC 27001

CACTUS

Contact Editage

Worldwide
request@editage.com
+1 877-334-8243
www.editage.com

Japan
submissions@editage.com
+81 03-6868-3348
www.editage.jp

Korea
submit-korea@editage.com
1544-9241
www.editage.co.kr

China
fabiao@editage.cn
400-005-6055
www.editage.cn

Brazil
contato@editage.com
0800-892-20-97
www.editage.com.br

Taiwan
submitjobs@editage.com
02 2657 0306
www.editage.com.tw
